# Primary Angle Closure and Sequence Variants within MicroRNA Binding Sites of Genes Involved in Eye Development

**DOI:** 10.1371/journal.pone.0166055

**Published:** 2016-11-08

**Authors:** Haihong Shi, Junfang Zhang, Rongrong Zhu, Nan Hu, Hong Lu, Mei Yang, Bai Qin, Jian Shi, Huaijin Guan

**Affiliations:** Eye Institute, Affiliated Hospital of Nantong University, Nantong, China; University of Massachusetts Medical School, UNITED STATES

## Abstract

**Purpose:**

The formation of primary angle closure (PAC) and primary angle closure glaucoma (PACG) is regulated by a tissue remodeling pathway that plays a critical role in eye development. MicroRNAs (miRNAs) are powerful gene expression regulators and may exert their effects on tissue remodeling genes. This study investigated the associations between gene variants (single-nucleotide polymorphism, SNP) in miRNA binding sites in the 3’-UTR region of genes involved in eye development and PAC.

**Methods:**

The sample consisted of 232 PAC subjects and 306 controls obtained from a population-based cohort in the Funing District of Jiangsu, China. The markers include 9 SNPs in the *COL11A1*, *PCMTD1*, *ZNRF3*, *MTHFR*, and *ALPPL2* genes respectively. SNP genotyping was performed with a TaqMan-MGB probe using an RT-PCR system.

**Results:**

Of the 9 SNPs studied, the frequency of the minor A allele of *COL11A1* rs1031820 was higher in the PAC group than in the control group in allele analysis (p = 0.047). The genotype analysis indicated that *MTHFR* rs1537514 is marginally associated with PAC (p = 0.014). The CC genotype of rs1537514 was present solely in the PAC group. However, the differences lost significance after Bonferroni correction.

**Conclusion:**

Our study reveals a possible association of *COL11A1* and *MTHFR* with PAC in the Han Chinese population. These results will contribute to an improved understanding of the genetic basis of PACG.

## Introduction

Glaucoma is the leading cause of irreversible blindness worldwide. Clinically, primary glaucoma is divided into primary open-angle glaucoma and primary angle closure glaucoma (PACG), depending on the anatomical characteristics of the anterior chamber angle. Asian populations are at higher risk of developing PACG than other ethnic groups [1], and it is estimated that PACG affects as many as 4.5 million people in China, constituting half the world's cases of PACG [1].

Eyes with PACG possess characteristic anatomical features, such as smaller corneal diameter (both horizontal and vertical from 'white-to-white'), steeper corneal curvature, shallower anterior chamber, thicker and more anteriorly positioned lens, and shortened eye ball, often accompanied by hyperopic refraction error [2]. Because ocular anatomic features are predisposing factors for PACG, the genes related to the regulation of axial length (AL) and the structural remodeling of connective tissues may contribute to the development of PACG. Several tissue remodeling genes, including *frizzled-related protein* (*MFRP*) [3–5], *extracellular matrix metalloprotease-9* (*MMP-9*) [6–8], *methylenetetrahydrofolatereductase* (*MTHFR*) [9], and *heat shock protein 70* (*HSP70*) [5,10], have been associated with PACG. However, the above findings remain controversial and were unable to be replicated in different ethnic groups. Apart from the above candidate gene association studies, some genome-wide association study (GWAS) added substantial data to the genetics of PACG. A GWAS identified three new susceptibility loci for PACG, including rs11024102 in *PLEKHA7*, rs3753841 in *COL11A1*, and rs1015213 in *PCMTD1-ST18* [11]. The mechanism of action of these genes in the pathogenesis of PACG is unknown and likely acts by affecting eye development [11,12]. Another GWAS confirmed the association at these three loci and found significant association of five additional loci with PACG [13]. Nongpiur et al. conducted a two-staged GWAS and found that a genetic variant within *ABCC5* gene may increase the risk of having PACG by shaping the anterior chamber depth (ACD) [14].

The above-described SNPs associated with PACG all occur in the coding, promoter or intron regions of the genes. While some 3'-untranslated (3'-UTR) SNPs participate in fibrotic remodeling diseases [15,16], no SNPs in microRNA (miRNA) binding sites in the 3'-UTR of any genes have been reported as associated with PACG or primary angle closure (PAC). miRNAs are short noncoding RNAs and key regulators of protein-mediated gene expression [17]. Evidence suggests that miRNAs play essential roles in controlling cellular processes, such as cell proliferation, apoptosis and differentiation [18]. miRNAs repress target mRNA expression by binding the miRNA regulatory elements located in the 3'-UTR of mRNAs. Thus, SNPs in the 3-'UTR highly influence the binding efficiency of miRNAs and mRNA, causing gain or loss of function and thereby regulating gene expression [19].

We hypothesized that SNPs in the miRNA binding sites of genes involved in eye development are associated with PACG or PAC. PAC shares the same anatomical features with PACG but does not present a glaucomatous optic neuropathy. Considering the obvious anatomic features in PAC whose pathogenesis might be of less confounding than those of PACG, we studied the association between SNPs in miRNA binding sites of the genes described above and other eye development-regulating genes, such as *ALPPL2* and *ZNRF3* [20] with PAC instead of PACG. To our knowledge, this is the first report of SNPs in miRNA binding sites associated with PAC in a Han Chinese population.

## Materials and Methods

### Study subjects

The study was approved by the Ethics Committee of the Affiliated Hospital of Nantong University and complied with the tenets of the Declaration of Helsinki. Each participant was fully informed of the purpose and procedures involved in the study and signed the informed consent form. The general demographic information of the participants is listed in Table 1. All participants were recruited using a cluster random sampling strategy from a population-based cohort conducted in the Funing District of Jiangsu, China. Of the 6,032 people aged 50 years and above screened, 232 subjects met the criteria of inclusion and exclusion of PAC defined for this study. We included 306 controls within the same communities and matched for sex and age with PAC subjects. The participants were all unrelated and self-identified as Han Chinese. There was no difference between the control group and the PAC group in terms of gender, age and major systemic diseases provenances.

**Table 1:** Demographic characteristics of study participants.

Demographic features	Control n (%)	PAC n (%)	P
Female	248 (81.05)	191 (82.33)	0.70
Male	58 (18.95)	41(17.67)	
Mean age (year) ± SD	65.08 ± 7.53	64.84 ± 8.59	0.74
Age range	50–85	50–83	
Hypertension	66 (19.64)	46 (19.83)	0.69
Diabetes	24 (7.36)	20 (8.6)	0.76
Cerebrovascular disease	10 (3.27)	4 (1.72)	0.41

This sample size has an 80% power to detect 5.8% confidence interval from a 10% allele frequency of a SNP based on the null hypothesized, regardless of multiple testing correction. The two SNPs of interest have the allele frequencies of 6.0% (rs1031820) and 8.4% (rs1537514) in the control group, thus the sample size has an 80% power to detect 2.6% and 5% confidence interval respectively.

The participants underwent thorough ocular examination that included best-corrected visual acuity, anterior segment photography, Goldmann applanation tonometry, fundus examination, optic disc photography, visual field, objective refraction and subjective refraction. The depth of the peripheral anterior chamber was evaluated using the Van Herick technique [21]. Participants with a peripheral chamber depth less than one-third the corneal thickness were invited for gonioscope, A scan ultrasonography and ultrasound biomicroscopy (UBM, SW-3200S, SUOER, Tianjin, China) examinations. UBM examination was conducted under light and dark conditions and in eight positions. The detailed protocol for gonioscopy and UBM was adopted from Barkana et al. [22].

PAC was defined according to the International Society of Geographical and Epidemiologic Ophthalmology (ISGEO) classification reported by Foster et al. [23] as follows: 1) Either eye shows the presence of an occluded angle (at least 180° of closed angle in which the trabecular meshwork is not visible on gonioscopy or iris apposition to the trabecular meshwork greater than 180° on UBM); 2) There are no sign of glaucomatous optic neuropathy or peripheral visual loss; 3) There are no sign of secondary angle closure; and 4) There are no previous ocular surgery or laser therapy. The clinical features of the PAC subjects are listed in Table 2.

**Table 2:** Clinical features of PAC subjects.

	Right eye	Left eye	Total eyes
Axial length (mm)	22.17 ± 0.83	22.17 ± 0.82	22.17 ± 0.83
ACD (mm)	2.49 ± 0.29	2.45 ± 0.30	2.47 ± 0.29
Refractive (diopter)	0.53 ± 1.85	0.68 ± 1.87	0.58 ± 1.84
Tonometry (mmHg)	15.18 ± 4.31	15.78 ± 4.46	15.52 ± 4.39

The criteria for enrollment of the control group were as follows: 1) peripheral chamber depth more than one-third the corneal thickness; 2) normal optic nerve heads with cup-to-cup ratio less than 0.5; 3) intraocular pressure less than 21 mmHg; 4) normal visual field; 5) no family history of glaucoma; 6) no ophthalmic diseases except slight cataract; and 7): refractive error less than three diopters.

### SNP selection

The initial selection of selected 3’-UTR SNPs were from 8 genes related to genetic susceptibility of PACG (*COL11A1*, *PCMTD1*, *PLEKHA7*, *HSP70*, *eNOS*, *MMP9*, *MTHFR*, *MFRP*) and 9 genes involved in eye development (*ZNRF3*, *ALPPL2*, *RSPO1*, *C3orf26*, *LAMA2*, *GJD2*, *CD55*, *MIP*, *ZC3H11B*) based on the literatures. The 3’-UTR SNPs to be investigated have minor allele frequencies (MAF) greater than 5% in Chinese Han populations, according to the HapMap database (http://hapmap.ncbi.nlm.nih.gov/, in the public domain), and small free energy (a parameter for nucleic acids binding affinity), referenced to the miRNA SNP 2.0 database (http://www.bioguo.org/miRNASNP/). After screening with these bioinformatics tools, we finally selected 9 3’-UTR SNPs in 5 genes that fell into the defined criteria, which included *COL11A1*, *PCMTD1*, *MTHFR*, *ZNRF3* and *ALPPL2*. Details of the tested SNPs are listed in Table 3. Their functions were summarized in the discussion section.

**Table 3:** SNPs tested and assay information from the NCBI and miRNA databases.

Gene	SNP	Ancestral allele	Assay ID	miRNA	Function	Chr site
*COL11A1*	rs9659030	T	C_29689555_10	hsa-miR-495	gain	Chr.1: 102876836
*PCMTD1*	rs6768	T	C_8891791_10	hsa-miR-217	gain	Chr.8: 51817881
				hsa-miR-297	loss	
*ZNRF3*	rs7290117	C	C_44007078_10	hsa-miR-1250	loss	Chr.22: 29054868
				hsa-miR-520f	gain	
*ZNRF3*	rs3178915	G	C_2479939_10	hsa-miR-4312	loss	Chr.22:29057039
*ZNRF3*	rs4823006	A	C_2479935_10	hsa-miR-331-3p	gain	Chr.22: 29055683
*ZNRF3*	rs2179129	A	C_2479934_10	hsa-miR-936	gain	Chr.22:29054935
*ALPPL2*	rs1009934	C	C_11531521_10	hsa-miR-3201	loss	Chr.22:29057205
*MTHFR*	rs1537514	C	C_8861307_10	hsa-miR-518a-5p	loss	Chr.1:11788011
				hsa-miR527		
				hsa-miR-596		
*COL11A1*	rs1031820	G	C_3087047_10	hsa-miR-107	gain	Chr.1:102877914
				hsa-miR-103		
				hsa-miR-4310		

### Genotyping

Genomic DNA was extracted from the peripheral blood of each individual using the Qiagen Blood DNA Mini Kit (Qiagen, Valencia, CA), according to the manufacturer’s instructions, and stored at -20°C.

Genotyping was conducted with the TaqMan genotyping assay (Applied Biosystems, Foster City, CA, USA) using the Real-time PCR 7500 system (Applied Biosystems, Foster City, CA, USA). The PCR procedure is described in our previous publication [5].

### Statistical analysis

Statistical analysis was performed with SPSS version 15.0 software. Differences in age and gender between PAC subjects and controls were assessed using the T-test and χ^2^ test, respectively. Hardy-Weinberg equilibrium (HWE) was tested using the χ^2^ test. The frequencies of genotypes and alleles were evaluated using the χ^2^ test or Fisher’s exact test. P values < 0.05 were considered statistically significant. In addition, logistic regression analysis was performed to calculate the odds ratio (OR) and 95% confidence interval (95% CI). If any positive association was found in the initial analysis, Bonferroni correction was performed. Three models of genotype are presented: the general model, the dominant model and the recessive model. Power analysis was conducted by Minitab 17 software.

## Results

The call rates of all SNPs genotyped were >98% and the call accuracies were 100% based on a 10% randomly selected sample. All 9 SNPs conformed to HWE (p>0.05) except for *MTHFR* rs1537514 in the PAC group.

Of the 9 SNPs tested, only COL11A1 rs1031820 showed a marginal association with PAC in allele analysis. The frequency of the minor A allele of rs1031820 was higher in the PAC group than in the control group (p = 0.047) and conferred an OR of 1.59 (95%CI: 1.00–2.53), indicating a harmful role of the SNP in developing PAC (Table 4). In the genotype analysis, the CC genotype of *MTHFR* rs1537514 was solely present in the PAC group. The differences regarding the genotypes between PAC and control were statistically significant (p = 0.014) and conferred an OR of 14.89 in recessive model, indicating its harmful roles in developing PAC (Table 5). However, these differences did not remain statistically significant after Bonferroni correction. The remaining 7 SNPs showed no differences in the distribution of genotypes and allele frequencies between the two groups.

**Table 4:** Allele frequency of SNPs in control and PAC subjects.

Gene	SNP(Minor/Major)	Call rate (%)	Allele distribution Minor/Major (%)		*P*	OR(95% CI)
			Control	PAC		
*COL11A1*	rs9659030(C/T)	98.9	159/447 (26.2)	108/350 (23.6)	0.323	0.87 (0.65–1.15)
*PCMTD1*	rs6768 (C/T)	99.6	118/490 (19.4)	82/382 (17.7)	0.469	0.89 (0.65–1.22)
*ZNRF3*	rs7290117 (T/C)	99.3	36/572 (5.9)	19/441(4.1)	0.190	0.68 (0.39–1.21)
*ZNRF3*	rs3178915 (A/G)	98.1	214/398 (39.0)	204/252(44.7)	0.061	1.27 (0.99–1.62)
*ZNRF3*	rs4823006 (A/G)	99.4	270/340 (44.3)	219/241(47.6)	0.277	1.14 (0.90–1.46)
*ZNRF3*	rs2179129 (G/A)	98.5	285/319 (47.2)	190/266(41.7)	0.074	0.80 (0.63–1.02)
*ALPPL2*	rs1009934(G/C)	98.7	146/460 (24.1)	114/342(25.0)	0.734	1.05 (0.79–1.39)
*MTHFR*	rs1537514(C/G)	98.5	51/553 (8.4)	38/418(8.3)	0.949	0.99 (0.64–1.53)
*COL11A1*	rs1031820(A/G)	98.7	36/568 (6.0)	42/416(9.2)	0.047	1.59 (1.00–2.53)

All HWE *p* values > 0.05 except MTHFRrs1537514 in PAC.

**Table 5:** Genotype frequency of SNPs in control and PAC subjects.

Gene	SNP	Genotype distribution n (%)			General *P* value	Dominant		Recessive	
			Control	PAC		*p*	OR(95% CI)	*p*	OR(95% CI)
*COL11A1*	rs9659030	TT	164(54.1)	136(59.4)	0.450	0.226	0.81(0.57–1.14)	0.98	0.99(0.50–1.98)
		TC	119(39.3)	78(34.1)					
		CC	20(6.6)	15(6.6)					
*PCMTD1*	rs6768	TT	160(52.3)	133(57.6)	0.474	0.94	0.65(0.57–1.34)	0.22	0.55(0.21–1.45)
		TC	116(37.9)	79(34.2)					
		CC	30(9.8)	19(8.2)					
*ZNRF3*	rs7290117	CC	270(88.8)	211(91.7)	0.310	0.26	0.72(0.40–1.29)	0.51	0.26(0.01–5.50)
		CT	32(10.5)	19(8.3)					
		TT	2(0.7)	0(0.0)					
*ZNRF3*	rs3178915	GG	114(38.0)	70(30.7)	0.172	0.08	1.38(0.96–1.99)	0.21	1.33(0.85–2.08)
		GA	138(46.0)	112(49.1)					
		AA	48(16.0)	46(27.4)					
*ZNRF3*	rs4823006	GG	96(31.5)	63(27.4)	0.544	0.31	1.22(0.83–1.78)	0.46	1.17(0.77–1.77)
		GA	148(48.5)	115(50.0)					
		AA	61(20.0)	52(22.6)					
*ZNRF3*	rs2179129	AA	78(25.8)	76(33.3)	0.157	0.06	0.70(0.48–1.02)	0.30	0.79(0.50–1.24)
		AG	163(54.0)	114(50.0)					
		GG	61(20.2)	38(16.7)					
*APPL2*	rs1009934	CC	176(58.1)	127(55.7)	0.776	0.58	1.10(0.78–1.56)	0.79	0.90(0.44–1.87)
		CG	108(35.6)	88(38.6)					
		GG	19(6.3)	13(5.7)					
*MTHFR*	rs1537514	GG	251(83.1)	195(85.5)	0.014	0.45	0.83(0.52–1.34)	0.01	14.89(0.82–270.80)
		GC	51(16.9)	28(12.3)					
		CC	0(0.0)	5(2.2)					
*COL11A1*	rs1031820	GG	266(88.1)	189(82.5)	0.076	0.07	1.51(0.96–2.55)	0.19	6.65(0.32–139.30)
		GA	36(11.9)	38(16.6)					
		AA	0(0.0)	2(0.9)					

All *p* values > 0.05 after Bonferroni correction.

## Discussion

To our knowledge, this was the first report from a population-based study to investigate the association of eye development genes with PAC. The design of a population-based study can reduce the sample selection bias often present in hospital-based case-control studies. Furthermore, this study was the first to discuss the association of SNPs in miRNA binding sites with PAC in a Han Chinese population. Our results showed that rs1537514 in the *MTHFR* gene and rs1031820 in the *COL11A1* gene are nominally associated with PAC, despite the loss of significance after rigorous Bonferroni correction.

Hyperhomocysteinemia is involved in alterations of the extracellular matrix and the structural remodeling of connective tissues [24,25]. Hyperhomocysteine may affect the overall anterior segment structure and may be involved in enhancing the attachment of the trabecular meshwork to the iris during development and aging [9]. The rs1801133 polymorphism in *MTHFR* gene leads to reduced enzyme activity and mild hyperhomocysteinemia, induces collagen and α-actin expression and causes extracellular matrix remodeling [8]. Michael et al. found that *MTHFR* rs1801133 was associated with PACG in a Pakistani population [9]. In their study, the minor TT genotype was present solely in the PACG group. Interestingly in our study, the wild-type CC genotype of rs1537514 was present solely in the PAC group, although the allelic frequency was similar in the control and PAC groups. When the C allele mutates to a G allele, the corresponding miRNAs cause loss-of-function and the loss of inhibition, while the wild-type CC genotype causes the corresponding miRNAs to bind to the 3'-UTR and leads to mRNA degradation or inhibition of translation, thereby down-regulating *MTHFR* expression and hyperhomocysteinemia. Our results indicated that the wild-type CC genotype of rs1537514 may contribute to PAC by negatively regulating *MTHFR* gene expression.

*COL11A1*and *PCMTD1* are genetic risk factors for PACG based on a GWAS [11]. The *COL11A1* gene codes for one of the two α-chains of type XI collagen, a minor fibril-forming collagen that controls fibril growth, diameter, and assembly of major collagens. This gene is expressed primarily in the articular cartilage and the ocular vitreous [26], as well as the trabecular meshwork [27]. Mutations in *COL11A1* cause disorders characterized by midfacial hypoplasia, sensorineural-hearing deficit, and nonprogressive axial myopia [28], such as Marshall syndrome, Stickler syndrome, and Stickler-like syndrome. In our present study, the frequency of the A allele of *COL11A1* rs1031820 was higher in the PAC group than in the control group. According to the miRNA SNP database, mutation in this 3'-UTR causes the corresponding miRNA to undergo gain of function and enhances its inhibition, thereby down-regulating *COL11A1* expression. This expression may be important in regulating drainage of the aqueous humor. For *PMCD1*rs6768, there were no significant differences between the two groups in regards to genotype frequency and alleles.

Recently, a GWAS meta-analysis identified 9 AL loci, 5 of which were associated with refraction in 18 independent cohorts [20]. However, three well-known loci identified in the GWAS on PACG were not associated with AL in this meta-analysis [11]. *ZNRF3* encodes proteins that are directly involved in the Wnt signaling pathway, the most significant pathway in vertebrate eye development [29]. Overexpression of a dominant-negative variant of human *ZNRF3* in zebrafish embryos induces small eyes or the absence of eyes [30]. *ALPPL2* was identified as associated with both AL and refraction, and the mRNA levels of *ALPPL2* show a two-fold difference in the sclera, retinal pigment epithelium, and neural retina in induced myopic eyes [31]. In our present study, none of the SNPs in *ZNRF3* and *ALPPL2* was significantly associated with PAC. Our results suggest that in addition to short AL and hyperopic refractive error, lens, iris, and anterior eye characteristics may serve as risk factors in PAC and PACG.

The study was nested in a perspective cohort of Jiangsu Eye Study and the sampling protocol was predetermined, which led to the limited PAC case available and caused potential underpowered in analysis. The profiling of ocular anatomic features particularly in control group was not be complete, which could result in possible missed opportunities. However, as the current available parameters, the data offered a potential for further studies.

In summary, our study reveals a nominal association of *MTHFR* rs1537514 and *COL11A1* rs1031820 with PAC in a Han Chinese population. The details of these miRNA-mRNA interactions in the context of gene variation and their roles in the pathogenesis of PACG require further study.

## Supporting Information

S1 File Genotype of 9 SNPs in control and PAC subjects. (XLS)

## References

[1] Foster PJ, Johnson GJ. Glaucoma in China: how big is the problem? Br J Ophthalmol. 2001;85: 1277–1282. 10.1136/bjo.85.11.1277 11673287 PMC1723754

[2] Sihota R, Lakshmaiah NC, Agarwal HC, Pandey RM, Titiyal JS. Ocular parameters in the subgroups of angle closure glaucoma. Clin Experiment Ophthalmol. 2000;28: 253–258. 11021552 10.1046/j.1442-9071.2000.00324.x

[3] Aung T, Lim MC, Wong TT, Thalamuthu A, Yong VH, Venkataraman D, et al Molecular analysis of CHX10 and MFRP in Chinese subjects with primary angle closure glaucoma and short axial length eyes. Mol Vis. 2008;14: 1313–1318. 18648522 PMC2480479

[4] Wang IJ, Lin S, Chiang TH, Chen ZT, Lin LL, Hung PT, et al The association of membrane frizzled-related protein (MFRP) gene with acute angle-closure glaucoma—a pilot study. Mol Vis. 2008;14: 1673–1679. 18781223 PMC2532703

[5] Shi H, Zhu R, Hu N, Shi J, Zhang J, Jiang L, et al Association of frizzled-related protein (MFRP) and heat shock protein 70 (HSP70) single nucleotide polymorphisms with primary angle closure in a Han Chinese population: Jiangsu Eye Study. Mol Vis. 2013;19: 128–134. 23378726 PMC3559095

[6] Aung T, Yong VH, Lim MC, Venkataraman D, Toh JY, Chew PT, et al Lack of association between the rs2664538 polymorphism in the MMP-9 gene and primary angle closure glaucoma in Singaporean subjects. J Glaucoma. 2008;17: 257–258. 10.1097/IJG.0b013e31815c3aa5 18552608

[7] Cong Y, Guo X, Liu X, Cao D, Jia X, Xiao X, et al Association of the single nucleotide polymorphisms in the extracellular matrix metalloprotease-9 gene with PACG in southern China. Mol Vis. 2009;15: 1412–1417. 19633731 PMC2714774

[8] Wang IJ, Chiang TH, Shih YF, Lu SC, Lin LL, Shieh JW, et al The association of single nucleotide polymorphisms in the MMP-9 genes with susceptibility to acute primary angle closure glaucoma in Taiwanese patients. Mol Vis. 2006;12: 1223–1232. 17110919

[9] Michael S, Qamar R, Akhtar F, Khan WA, Ahmed A. C677T polymorphism in the methylenetetrahydrofolate reductase gene is associated with primary closed angle glaucoma. Mol Vis. 2008;14: 661–665. 18385801 PMC2277437

[10] Ayub H, Khan MI, Micheal S, Akhtar F, Ajmal M, Shafique S, et al Association of eNOS and HSP70 gene polymorphisms with glaucoma in Pakistani cohorts. Mol Vis. 2010;16: 18–25. 20069064 PMC2805420

[11] Vithana EN, Khor CC, Qiao C, Nongpiur ME, George R, Chen LJ, et al Genome-wide association analyses identify three new susceptibility loci for primary angle closure glaucoma. Nat Genet. 2012;44: 1142–1146. 10.1038/ng.2390 22922875 PMC4333205

[12] Day AC, Luben R, Khawaja AP, Low S, Hayat S, Dalzell N, et al Genotype-phenotype analysis of SNPs associated with primary angle closure glaucoma (rs1015213, rs3753841 and rs11024102) and ocular biometry in the EPIC-Norfolk Eye Study. Br J Ophthalmol. 2013;97: 704–707. 10.1136/bjophthalmol-2012-302969 23505305 PMC4624259

[13] Khor CC, Do T, Jia H, Nakano M, George R, et al (2016) Genome-wide association study identifies five new susceptibility loci for primary angle closure glaucoma. Nat Genet 48: 556–562. 10.1038/ng.3540 27064256

[14] Nongpiur ME, Khor CC, Jia H, Cornes BK, Chen LJ, et al (2014) ABCC5, a gene that influences the anterior chamber depth, is associated with primary angle closure glaucoma. PLoS Genet 10: e1004089 10.1371/journal.pgen.1004089 24603532 PMC3945113

[15] Vettori S, Gay S, Distler O. Role of MicroRNAs in fibrosis. Open Rheumatol J. 2012;6: 130–139. 10.2174/1874312901206010130 22802911 PMC3396185

[16] Patel V, Noureddine L. MicroRNAs and fibrosis. Curr Opin Nephrol Hypertens. 2012;21: 410–416. 10.1097/MNH.0b013e328354e559 22622653 PMC3399722

[17] Bartel DP. MicroRNAs: genomics, biogenesis, mechanism, and function. Cell. 2004;116: 281–297. 14744438 10.1016/s0092-8674(04)00045-5

[18] Chen CZ, Li L, Lodish HF, Bartel DP. MicroRNAs modulate hematopoietic lineage differentiation. Science. 2004;303: 83–86. 10.1126/science.1091903 14657504

[19] Gong J, Tong Y, Zhang HM, Wang K, Hu T, Shan G, et al Genome-wide identification of SNPs in microRNA genes and the SNP effects on microRNA target binding and biogenesis. Hum Mutat. 2012;33: 254–263. 10.1002/humu.21641 22045659

[20] Cheng CY, Schache M, Ikram MK, Young TL, Guggenheim JA, Vitart V, et al Nine loci for ocular axial length identified through genome-wide association studies, including shared loci with refractive error. Am J Hum Genet. 2013;93: 264–277. 10.1016/j.ajhg.2013.06.016 24144296 PMC3772747

[21] Foster PJ, Devereux JG, Alsbirk PH, Lee PS, Uranchimeg D, Machin D, et al Detection of gonioscopically occludable angles and primary angle closure glaucoma by estimation of limbal chamber depth in Asians: modified grading scheme. Br J Ophthalmol. 2000;84: 186–192. 10.1136/bjo.84.2.186 10655196 PMC1723375

[22] Barkana Y, Dorairaj SK, Gerber Y, Liebmann JM, Ritch R. Agreement between gonioscopy and ultrasound biomicroscopy in detecting iridotrabecular apposition. Arch Ophthalmol. 2007;125: 1331–1335. 10.1001/archopht.125.10.1331 17923539

[23] Foster PJ, Buhrmann R, Quigley HA, Johnson GJ. The definition and classification of glaucoma in prevalence surveys. Br J Ophthalmol. 2002;86: 238–242. 11815354 10.1136/bjo.86.2.238 PMC1771026

[24] Mujumdar VS, Tummalapalli CM, Aru GM, Tyagi SC. Mechanism of constrictive vascular remodeling by homocysteine: role of PPAR. Am J Physiol Cell Physiol. 2002;282: C1009–C1015. 10.1152/ajpcell.00353.2001 11940516

[25] Tyagi SC. Homocysteine redox receptor and regulation of extracellular matrix components in vascular cells. Am J Physiol. 1998;274: C396–C405. 9486129 10.1152/ajpcell.1998.274.2.C396

[26] Annunen S, Korkko J, Czarny M, Warman ML, Brunner HG, Kaariainen H, et al Splicing mutations of 54-bp exons in the COL11A1 gene cause Marshall syndrome, but other mutations cause overlapping Marshall/Stickler phenotypes. Am J Hum Genet. 1999;65: 974–983. 10.1086/302585 10486316 PMC1288268

[27] Michael I, Shmoish M, Walton DS, Levenberg S. Interactions between trabecular meshwork cells and lens epithelial cells: a possible mechanism in infantile aphakic glaucoma. Invest Ophthalmol Vis Sci. 2008;49: 3981–3987. 10.1167/iovs.08-1674 18469193

[28] Kahler RA, Yingst SM, Hoeppner LH, Jensen ED, Krawczak D, Oxford JT, et al Collagen 11a1 is indirectly activated by lymphocyte enhancer-binding factor 1 (Lef1) and negatively regulates osteoblast maturation. Matrix Biol. 2008;27: 330–338. 10.1016/j.matbio.2008.01.002 18280717 PMC2431459

[29] Fuhrmann S. Wnt signaling in eye organogenesis. Organogenesis. 2008;4: 60–67. 19122781 10.4161/org.4.2.5850 PMC2613311

[30] Hao HX, Xie Y, Zhang Y, Charlat O, Oster E, Avello M, et al ZNRF3 promotes Wnt receptor turnover in an R-spondin-sensitive manner. Nature. 2012;485: 195–200. 10.1038/nature11019 22575959

[31] Barathi VA, Beuerman RW, Schaeffel F. Effects of unilateral topical atropine on binocular pupil responses and eye growth in mice. Vision Res. 2009;49: 383–387. 10.1016/j.visres.2008.11.005 19059278

